# Enhanced cognitive performance after multiple adaptations to visuomotor transformations

**DOI:** 10.1371/journal.pone.0274759

**Published:** 2022-09-21

**Authors:** Gerd Schmitz

**Affiliations:** Institute of Sports Science, Leibniz University Hannover, Hannover, Germany; L V Prasad Eye Institute, INDIA

## Abstract

Several studies reported that adaptation to a visuomotor transformation correlates with the performance in cognitive performance tests. However, it is unclear whether there is a causal relationship between sensorimotor adaptation and cognitive performance. The present study examined whether repeated adaptations to double steps and rotated feedback increase cognitive performance assessed by neuropsychological tests in a pre-post design. The participants of the intervention group adapted in 24 sessions their hand movements to visuomotor transformations with increasing size. Pre-post changes were significantly larger in the intervention group than in a control group without training. This result suggests a causal relationship between sensorimotor adaptation training and cognitive performance.

## Introduction

A fundamental human ability is the ability to adapt to changing environments. Sensorimotor adaptation seems to involve motor processes and higher-level cognitive processes, and thus it seems to be based on the interaction of several hierarchies within the central nervous system. Sensorimotor adaptation is typically investigated in experiments on hand or arm movements [1] as these movement types “represent an intermediate level of behavior that embodies both low-level motor execution and higher-level cognition” [2, p. 1]. Accordingly, it was reported that participants adapt faster when they are aware of the mismatch between the perceived and the expected outcome of their action (prediction or performance error), if they have a good declarative and spatial working memory, or have well-developed abilities related to divergent thinking, figural fluency, sustained attention and divided attention [3–12]. Other results have shown that participants who outperform others in tests measuring selective or divided attention, decision making, visuomotor skills, or have a lower tendency for perseverative behavior achieve better and more persistent results in motor adaptation tasks [8, 13–16]. Switching to another adaptation context, either by performing another movement task, using another effector, or adapting to a different sensorimotor transformation, is related to awareness and explicit knowledge about the transformation, cognitive inhibition, and mental flexibility [8, 14, 15, 17, 18]. These findings imply a functional relationship between cognitive control and sensorimotor adaptation abilities.

However, the question of a causal relationship between sensorimotor adaptation and cognitive performance remains unanswered. Most of the studies investigating this topic were cross-sectional. Only a few studies have investigated longitudinally whether cognitive training improves sensorimotor adaptation or vice versa. Anguera et al. [19] scrutinized whether a five-week working memory training improves sensorimotor adaptation abilities. Although the training improved working memory capacity, it did not improve sensorimotor adaptation performance. Nevertheless, adaptation was significantly degraded by short-term depletion of working memory resources, indicating that a certain level of working memory resources is required. Longitudinal studies investigating whether sensorimotor adaptation training improves cognitive abilities are lacking. However, reports on savings point in this direction [20, 21]. The term savings describes a phenomenon that occurs when participants perform an adaptation task for the second time: The adaptation rate increases from the first to the second adaptation session even if one year has passed [20]. Ruitenberg et al. [21] analyzed the brain activity during four adaptation sessions in three months. They found that the increased adaptation rates from the second to the fourth session coincide with increased activity in brain areas associated with cognitive processing. The results indicate that consecutive adaptation sessions engage and challenge cognitive processes, which were, in this case, related to action selection. Results from related research fields demonstrate an increase in spatial cognitive performance in blind individuals through memory-guided freehand drawing [22]. However, behavioral results referring to enhanced cognitive performance after adaptation are still lacking. The present study tested the hypothesis that consecutive adaptations to sensorimotor transformations significantly enhance cognitive performance. Post-pre-changes in cognitive performance tests were compared between an intervention and a passive control group to scrutinize cognitive performance enhancements. To specify possible causes for the cognitive improvements demonstrated in the group comparison, the adaptation data of the intervention group were additionally analyzed with significant post-pre changes of the cognitive tests as covariates.

The study design considers adaptation factors that have been associated with different cognitive abilities in cross-sectional studies: Adaptation at different time scales [14], adaptation to increasing angular transformations [8], and interference between successive adaptation tasks [15]. At the same time, a multifactorial design was expected to promote compliance to participate in the longitudinal experiment because it creates a relatively high degree of variety concerning the execution of the tasks.

The participants of the intervention group practiced four adaptation tasks, which required the learning of new visuomotor transformations. A visuomotor transformation represents a mismatch between how an action is performed and how action effects are visually perceived. A basic visuomotor transformation is already learned by operating a computer: a computer mouse moves in the horizontal plane, but the visual cursor moves in the vertical plane. The concurrent learning of multiple new angular visuomotor transformations is challenging because their learning interferes. However, extensive training reduces interference over time and concurrent learning becomes possible [17, 23]. In order to keep the task challenging, the discordance size of the angular transformation increased incrementally. It was hypothesized that the extensive training in four adaptation tasks with increasing discordance size improves cognitive performance. To test this hypothesis, 24 participants trained 24 times for about 30 minutes each in a home-based setting with an interactive computer program. Post-pre changes in cognitive performance were measured with neuropsychological tests.

## Materials and methods

19 women and 17 men participated in the study. An overall sample size of N = 24 for between-group comparisons (i.e., n = 12 per group) was calculated from a power analysis according to Faul et al. [24], considering the largest effect reported in the study from Schmitz et al. [8] (effect size: f = 0.62, level of significance: α = 0.05, power: 1-ß = 0.80). The sample size for the intervention group was doubled due to the additionally planned within-group analyses resulting in an overall sample size of N = 36; i.e., n = 24 for the intervention and n = 12 for the control group. The decision to double the sample size of the intervention group was made a priori. It was not based on a power analysis because the number of the potential covariates could not be determined a priori: Only variables that differ significantly between the intervention and the control group should be considered for the within-group comparisons in the intervention group. Thus, 15 females and 9 males performed visuomotor training (intervention group). Four females and 8 males belonged to a passive control group without intervention. The participants were on average 25.5 years old (standard deviation, SD: 5.9). Age did not differ significantly between the groups (t(34) = 1.42, p = 0.164, d = 0.50). The participants were free from overt neurologic or psychological impairments, and all gave their written informed consent to the study. All procedures were applied in accordance with the 1964 Helsinki declaration and its later amendments. The study protocol had been preapproved by the local ethics committee of the Leibniz University Hannover. Participation in the study was remunerated with 8 euros per hour.

### Cognitive performance tests

Before and after the sensorimotor adaptation training, all participants performed seven neuropsychological tests (paper-pencil-tests). The tests were administered and scored by an experimenter. Previous studies have shown that the outcomes of these tests correlate with the performance during adaptation to visuomotor transformations as described below:

1. Frankfurter Attentional Inventory 2 (FAIR 2). Four marginally differing symbols are presented in random order 640 times on two DIN A4 pages. Two of the four symbols represented target symbols. The participants draw a line under the symbols. As soon as they identify a target item, they draw the line into the corresponding symbol. The participants have to mark as many target symbols as possible within 6 minutes. The dependent variable K is interpreted as a measure of sustained attention [25]. A larger K reflects better performance. This parameter was a significant covariate for adaptation to increasing discordance sizes in one study [8]. Further studies reported the significance of attentional resources for adaptation (e.g. [13]).
2. Number Connection Test. The participants have to connect the numbers 1–90 on a DIN A 4 page in ascending order as quickly as possible. Each higher number is located in spatial proximity to the former number (directly or in the diagonal to the left or right, above or below). The performance depends on cognitive processing speed and decision-making abilities [26]. Furthermore, processing speed and decision time correlate with the performance during adaptation [14].
3. Trail Making Test (TMT): The participants have to connect numbers (task A) or alternatingly numbers and letters (task B) as quickly as possible. The symbols are randomly distributed on a DIN A4 page. The task is supposed to measure cognitive processing speed (task A, task B), visuospatial orientation as well as fluid cognitive abilities (task A, task B) and cognitive flexibility (task B) [27–30]. The performance in this test was a significant predictor of the interference between consecutive adaptations in [15]. Processing speed and flexibility were also significant predictors for adaptation [14].
4. Five-Point Test: The participants have to produce as many unique figures as possible within 3 minutes by connecting two to five points pre-printed in rectangles (40 rectangles per DIN A4 page). They are instructed to avoid repetitions. The number of unique designs is interpreted as a measure of figural fluency and divergent thinking, and the number of repetitions divided by the number of all designs as a measure for perseveration [28, 31, 32]. Both parameters are significant covariates for the adaptation to increasing sensorimotor discordances [8]. Perseveration is also a predictor of the generalization of adaptation and the interference between consecutive adaptations [15]. Moreover, divergent thinking abilities predict how fast participants adapt [12].
5. Stroop Test. The participants have to read aloud the words ‘blue’, ‘yellow’, ‘green’ and ‘red’ printed in black (task 1), to name the color of blue, yellow, green and red bars (task 2) and to name the print-color of words, whose word-meaning deviates from their print-color; for example, if the word ‘green’ is printed in yellow, the correct answer is ‘yellow’. Each task requires 72 responses. The dependent variable is performance time. Factor analyses indicate that the test performance primarily depends on action initiation (tasks 1 to 3), reading speed (task 1), nomination speed (task 2) and cognitive control of attention during interference (task 3) [33, 34]. The performance in this test is a significant predictor of the interference between consecutive adaptations [15].
6. Maze Test. The task is to move a pen from the center of a maze (Porteus-maze) to a target position at the outer edge of the maze as quickly as possible. Measured are performance time for a pseudo-maze without bifurcations and performance time for a maze with bifurcations. The maze-test requires visuomotor abilities (pseudo-maze, maze) as well as visuospatial planning and decision-making abilities (maze) [35]. The performance in this test is a significant predictor of the interference between consecutive adaptations [15].
7. Digit Span Forward. The participants have to repeat verbally a sequence of single digit numbers read out by the experimenter. The digits of a number are read out at one-second intervals. The participants have to hold the digits in consciousness until a number has been read out completely. After two trials with the same number of digits, the following trial contains a sequence including an additional digit. The test stops when both trials with the same number of digits are wrong. The number of correct answers is taken as a measure of verbal working memory [34]. The performance in this test is a significant predictor for the generalization of adaptation and the interference between consecutive adaptations [15]. Verbal working memory seems particularly important for fast adaptation processes [5].

The test order was randomized across participants, but the pre-test order was similar to the order of the post-tests within each participant. Pre- and post-tests were on average 79 days apart. This period did not differ significantly between the 24 participants who performed adaptation training and the 12 participants who served as controls (t(34) = 1.46, p = 0.153, d = 0.52).

### Visuomotor adaptation training

The participants of the intervention group conducted the visuomotor training at home on their own Notebook-PC. For this purpose, a user-friendly interactive computer program was developed. The experimenter installed the software on the participants’ Windows devices. Once started, the software automatically guided the participant through the training program. The participants only had to press the space bar to start a new episode. The author introduced the task and supervised each participant during the first adaptation session.

The participants of the intervention group performed 24 training sessions of about 30 minutes each at home. The software stored automatically all data (starting and ending time of the session, movement and target data, experimental conditions) in hidden files in anonymized form. Each participant manually documented the start and end of each training session. Random samples of the manual documentations were compared with the hidden electronic documentations of the training software for control purposes. In all cases, the manual documentations corresponded to the hidden log files.

**Fig 1:**
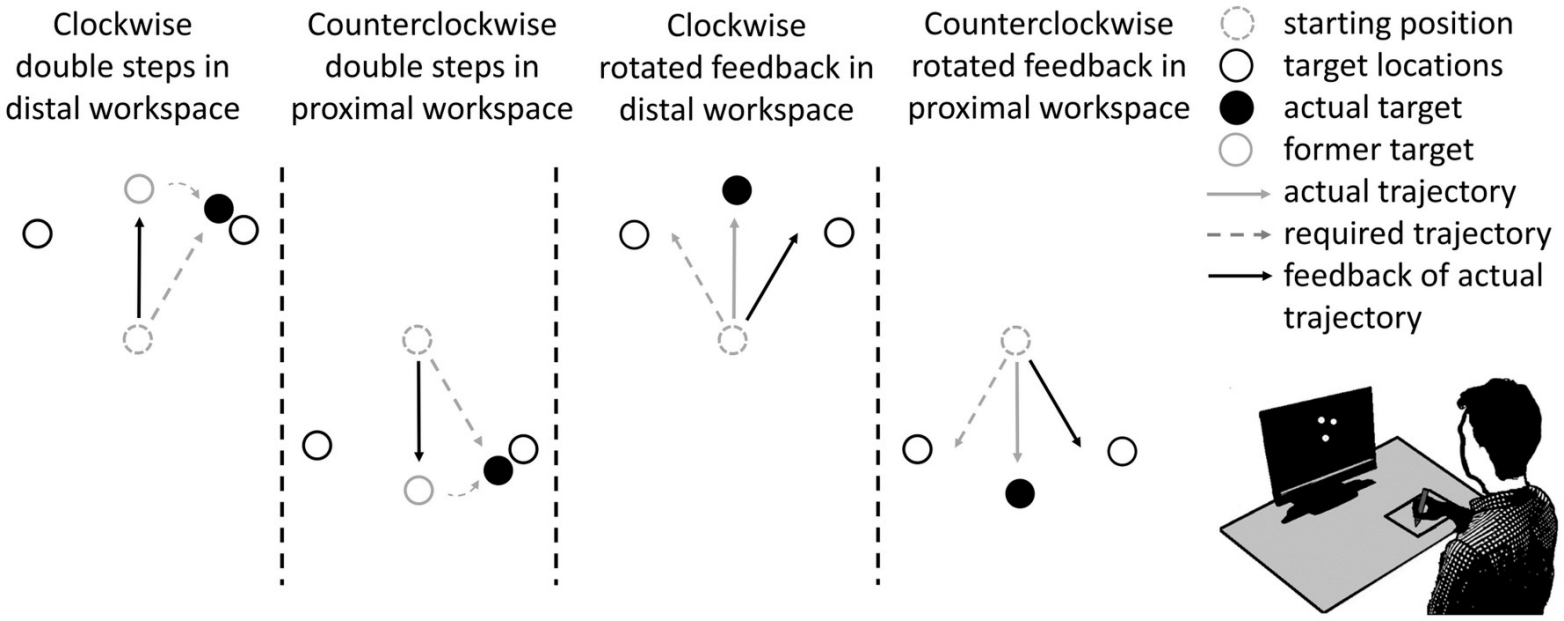
Adaptation tasks. Each participant adapted in the distal and the proximal workspace to double steps as well as to rotated feedback. Double steps represent target displacements at movement onset–here illustrated by a change from a former to the actual target. Clockwise double steps (here shown for the distal workspace) and counterclockwise feedback rotations (here shown for the proximal workspace) require clockwise movement trajectory modifications to reach the target. Counterclockwise double steps (here shown for the proximal workspace) and clockwise feedback rotations (here shown for the distal workspace) require counterclockwise trajectory modifications.

The participants used a digitizer tablet (Trust® Flex Design Tablet, 16.3 * 19.3 cm, resolution 2000 LPI, 120 Hz) as input device (Fig 1). They were instructed to locate it in front of the shoulder of their dominant hand in parallel with the notebook keyboard. The pen position on the tablet determined the cursor’s position on the screen. When the stylus moved 1 cm forward on the tablet, the cursor moved 1 cm upwards on the vertical screen. This basic visuomotor transformation is comparable to the input procedure with a computer mouse, and the participants reported being familiar with it.

The task required discrete reaching movements between a starting point at the center of the screen and six peripheral targets. The participants were instructed to move the cursor (0.5 cm radius) quickly and accurately in a straight line from the starting point to the current peripheral target. When the cursor reached the target position for a cumulative time of 750 ms, or in the trials without a cursor after 3000 ms, the target disappeared, and the participants moved the pen back to the center of the tablet. Reaching the center was indicated by a change in the color of the starting point on the screen. All targets had a radius of 0.5 cm and were placed on a single non-visible circle (radius of 5 cm). Three targets were positioned above (distal workspace) and three below the starting point (proximal workspace). In the distal workspace, the targets were presented at the directions 45°, 90° and 135°. In the proximal workspace, the targets were presented at the directions 225°, 270° and 315° (Fig 1). (0° is to the right of the center, and directions increase in a mathematically positive sense.) Thomas and Bock [36] have shown that the differentiation of a distal and proximal workspace allows the concurrent learning of otherwise interfering visuomotor transformations. In contrast, clockwise and counterclockwise adaptations of movement directions interfere when performed in the same workspace [15, 36, 37].

Each participant trained four adaptation tasks in a sequence. All participants started with the distal workspace and then switched to the proximal workspace. Fig 1 illustrates 30° clockwise double steps for the distal workspace requiring 30° clockwise rotations of movement trajectories. Double steps are characterized by target displacements at movement onset; i.e., when the movement exceeds a velocity threshold of 40 mm/s, the target steps from its original position to another position with the same distance but a different angle compared to the former position. Counterclockwise double steps requiring counterclockwise trajectory modifications are shown for the proximal workspace. 30° clockwise rotated feedback, which requires 30° counterclockwise rotations of movement trajectories, is shown for the distal workspace. Counterclockwise rotated feedback, requiring clockwise trajectory modifications, is shown for the proximal workspace. The present study applied both the rotated feedback method and the double step method to make the task varied for the participants. Former studies have shown that both methods achieve similar effects when adaptation is long enough and interfere when applied in the same workspace [8, 15, 38].

The order of the adaptation methods (double steps versus rotated feedback) and the directions of the trajectory modifications (clockwise versus counterclockwise) were balanced across participants. This procedure resulted in four subgroups with six participants each. That means that half of the participants adapted to double steps in the first two tasks, the other half to feedback rotations. Half of the participants adapted their trajectories in the first task clockwise and the second task counterclockwise. The other half adapted their trajectories in the first task counterclockwise and in the second task clockwise. In the third and fourth adaptation tasks, the other adaptation method was applied and the participants adapted in each workspace to the opposite direction compared to the first two adaptation tasks.

In summary, each participant performed four adaptation tasks. Since the order of the tasks varied between the participants, the first, second, third, and fourth adaptation tasks will be referred to in the following.

### Procedure of the visuomotor adaptation training

Each session started with the basic visuomotor transformation with feedback (baseline phase). Next came the visuomotor pre-test without feedback, in which the participants of the intervention group performed reaching movements without cursor feedback. The session was concluded with a visuomotor post-test without feedback, which was compared to the pre-test without feedback to detect possible aftereffects. The adaptation training was performed between both tests without feedback and is described in the following.

**Fig 2:**
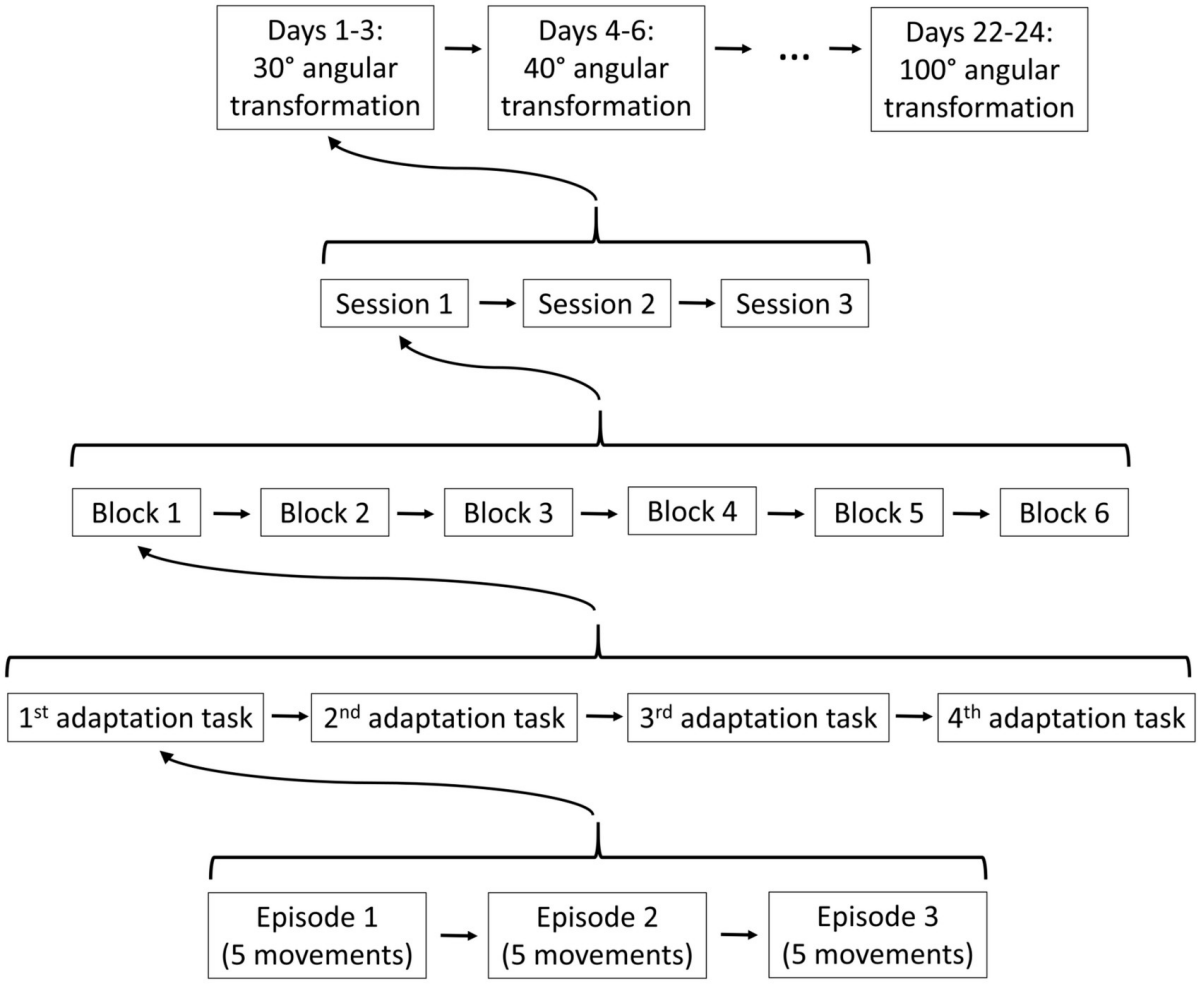
Experimental design. The participants of the intervention group trained for three episodes of five movements a first adaptation task, then trained for three episodes a second adaptation task and so on. The four adaptation tasks were practiced in six blocks, which constituted one session. One to three days later, they performed the next training session. Three sessions were trained with a given angular transformation before it increased by 10°.

Compared to the baseline phase, the adaptation tasks shown in Fig 1 represent new relationships between hand movement direction in the horizontal plane and the direction of the visual feedback on the screen (i.e., new angular transformations). As first shown by Smith et al. [39] for force-field adaptation, adaptation seems to proceed in multiple timescales. This finding has been confirmed for other types of adaptation, such as the learning of a new angular transformation or saccadic adaptation [40, 41]. The procedure for the adaptation training is shown in Fig 2: Each adaptation task was practiced for three episodes of five movements each. The sequence of four adaptation tasks constituted one block. One session consisted of the baseline phase, the visuomotor pre-test, six blocks and the visuomotor post-test. In the first session, the angular transformations had a size of 30°. It is well known that adaptation saturates after a few hundred trials. To provide significant adaptation stimuli throughout the intervention, the angular transformation increased after three sessions in 10°-steps from 30° to 100°. Two sessions were one to three days apart. The final assessment of cognitive performance (cognitive post-test) was performed two days after the last intervention.

### Data analysis

The learning of an angular visuomotor transformation occurs by adaptation of trajectory planning processes that remaps the movement vector, i.e., movement direction, but not the final positions of limb movements [42]. In the present study, the movement direction was measured as the angle between the target vector and the movement vector 100 ms after movement onset (initial movement direction). This assures that movements are not visually corrected because visual feedback needs longer latencies to become effective [43]. The decision to focus in the analysis on the initial movement direction and not on the end position is supported by findings from Schmitz [15], who showed that executive functions rather correlate with the adaptation of initial movement directions than with the adaptation of movement endpoints. Movement onset was defined as the point in time when the movement velocity exceeds 25 mm/s. The velocity threshold is an experience value, which has shown to be insensitive against small corrective movements of the pen around the starting point.

The median direction of five movements (one episode) was calculated and submitted to the statistical analyses. Normality distributions were tested with the Kolmogorov-Smirnov test. Pre-test values and post-pre-differences from the cognitive performance tests (cognitive post-pre-changes) were z-transformed and compared across groups by a two-way ANOVA with the between-subject factor ‘group’ (intervention / control) and the within-subject factor ‘test’ (cognitive performance test measures). In this ANOVA, only the main effect ‘group’ and the ‘test*group’ interaction but not the main effect ‘test’ were analyzed because the latter has no variance due to the z-transformations of the variables.

Furthermore, for the statistical analyses, the algebraic signs of the cognitive post-pre changes were inverted for correct patterns in the Five-Point Test, the variable K from the FAIR-2-Test and the number of correct answers from the Digit Span Test because positive signs reflect a performance increase, whereas in the other tests, negative signs reflect performance increases. For one participant, the post-pre-data of the Trail Making Test were missing. Therefore, two analyses were performed: One analysis with all tests but without this participant and a second analysis with all participants but without the TMT.

The performance during the baseline phase was compared across all sessions by a three-way ANOVA with the within-subject factors angular transformation (30°, 40°, 50°, 60°, 70°, 80°, 90°, 100°), session (1–3) and workspace (distal versus proximal). Visuomotor pre-post changes were analyzed by a four-way ANOVA with the within-subject factors phase (pre- versus post-test), angular transformation (30°-100°), session (1–3) and workspace (distal versus proximal).

The performance during the training of the angular transformations was analyzed by a six-way ANOVA with the within-subject factors angular transformation (30°-100°), session (1–3), block (1–6), workspace (distal versus proximal), order (first & second versus third & fourth adaptation task) and episode (1–3). To analyze covariations between sensorimotor learning and cognitive performance, each participant’s mean z-value of the pre-test as well as of the post-pre-changes in the cognitive performance tests were submitted as covariates to the ANOVA of the sensorimotor training. Partial eta-squared (ŋ^2^_p_) is reported as the effect size measure. Sphericity was tested with the Mauchly test. Huynh-Feldt adjustments were applied in case of its significance. The homogeneity of variances was analyzed with the Levene test. Post hoc comparisons were performed with the Newman-Keuls procedure.

Finally, a factor analysis based on the principal component method was calculated for the cognitive post-pre-changes. The varimax rotation was selected as rotation method. It creates a simple structure of the factors by maximizing the squared loadings per factor [44]. The selection criteria for variables were anti-image correlations larger than 0.5 and communalities larger than 0.7. Factors with eigenvalues larger than 1 were regarded as meaningful (Kaiser-Guttman-criterion). If the eigenvalue of a factor is smaller than 1, it is smaller than the variance of a single standardized variable. This factor is generally considered insignificant as it can no longer contribute to the data reduction [44]. The number of factors was chosen based on a visual inspection of Cattell’s scree plot. The minimal acceptable Kaiser-Meyer-Olkin-criterion was defined as 0.6. The stability of the factor structure was estimated according to the procedure suggested by Bortz and Schuster [44]. This procedure considers sample size and minimal factor loading taken into account when interpreting the factors. It yields a descriptive measure of stability, which should be ≥ 0.8.

## Results

### Baseline phase and visuomotor pre-post changes

**Fig 3:**
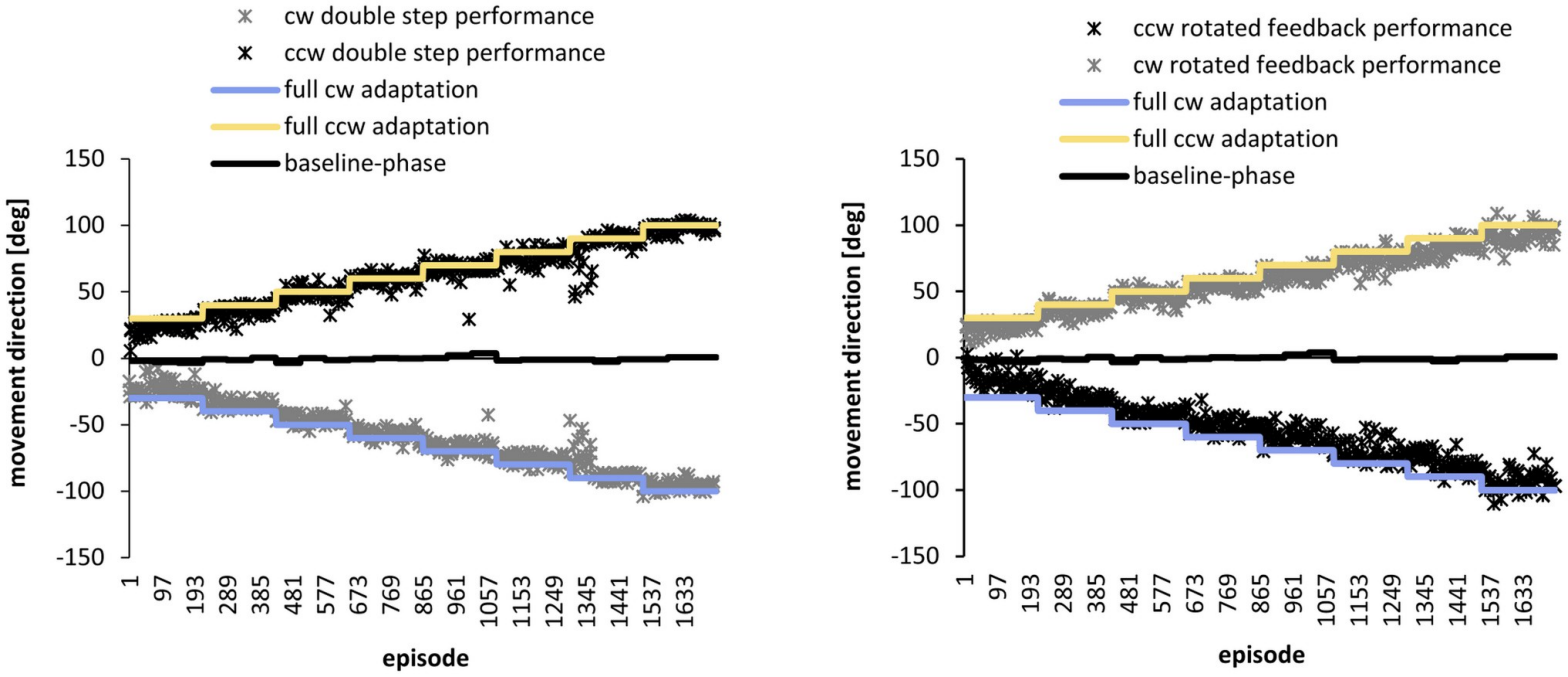
Performance of one participant. The participant adapted to clockwise (cw) double steps in the distal workspace, counterclockwise (ccw) double steps in the proximal workspace, counterclockwise rotated feedback in the distal workspace, and clockwise rotated feedback in the proximal workspace. Illustrated are the movement directions measured 100 ms after movement onset. Each dot represents the mean of one episode, i.e., of 5 movements. All episode means of the intervention are shown. The solid blue and orange lines represent the expected values for complete adaptation of movement directions to the angular transformations 30°-100°. The solid black line near the Abscissa represents the performance during the baseline phase.

Each session started with the baseline phase, in which the participants of the intervention group moved the stylus with a mean deviation of -0.46° (SD: 1.76°) to the targets. This value represents the initial movement direction, i.e., the angle between the target vector and the movement vector 100 ms after movement onset. Data from an exemplary participant are shown in Fig 3. Depicted are the mean initial movement directions of all episodes of this participant. The baseline performance did not change during the study. An ANOVA on the data of the baseline phase of all participants resulted in only one significant effect, a significant interaction between angular transformation and workspace (F(7,161) = 2.63, p = 0.013, ŋ^2^_p_ = 0.10): At the beginning of the sessions with the 80° angular transformations, the initial movement directions in the distal (-1.30°) and the proximal workspace (0.99°) differed significantly from each other (post-hoc test: p = 0.031). Other effects were not significant. The movement directions in the visuomotor pre- and post-test without feedback deviated from the target directions on average by 0.08° (SD: 1.27°). Neither the pre-post changes nor any other factors were significant. Thus, aftereffects could not be detected.

### Performance in the adaptation tasks

Fig 3 also shows the adaptation performance of the exemplary participant. Clockwise adaptation, reflected by negative movement directions, was required in response to clockwise double steps and counterclockwise feedback rotations. Counterclockwise adaptation, reflected by positive movement directions, was required in response to counterclockwise double steps and clockwise feedback rotations. The increasing values at each of the four adaptation tasks indicate that the participant adapted to all of them. Furthermore, the data series do not overlap but diverge instead, indicating that this participant was able to switch from clockwise to counterclockwise adaptation and vice versa. In the subsequent analyses, the algebraic signs of the movement directions during clockwise adaptation were inverted to allow comparability between subsequent adaptation tasks.

**Fig 4:**
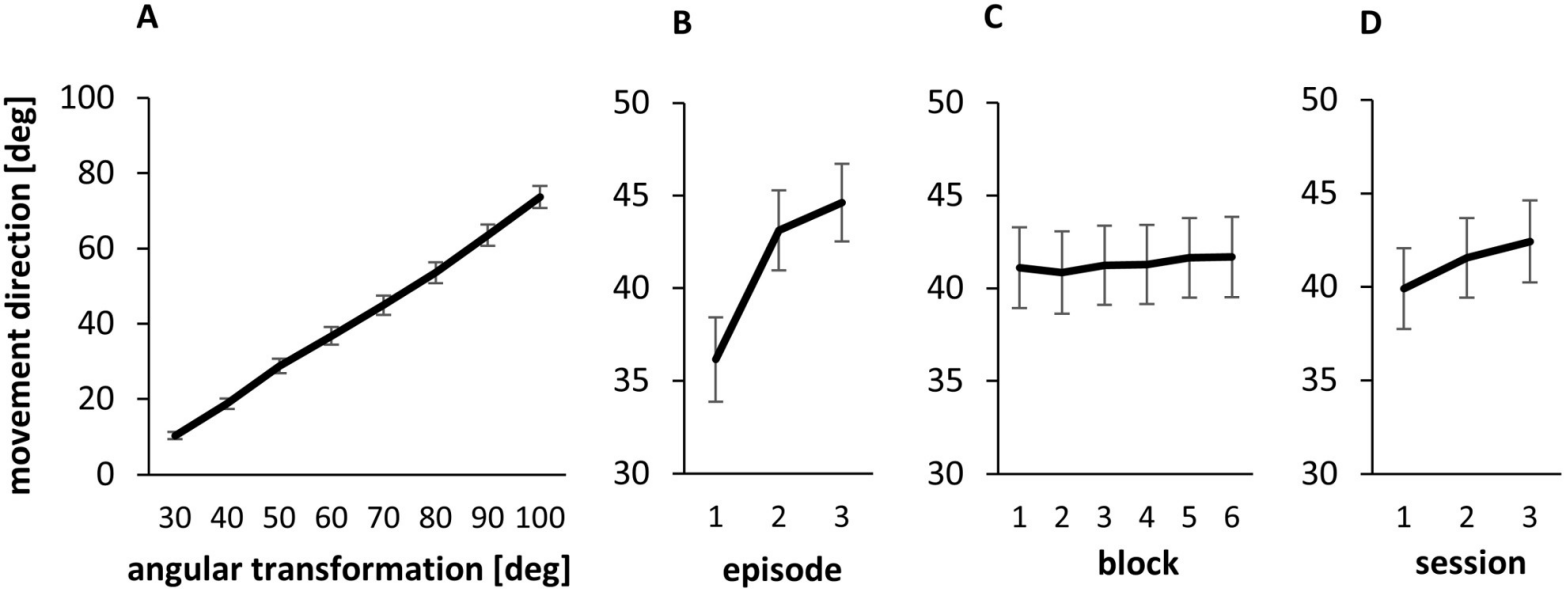
Mean performance averaged across the four adaptation tasks. 0° represents the unadapted expectation. A: The size of the angular transformation corresponds to the expected value for complete adaptation. B-D: 65° represents the expected value for complete adaptation. B-D show the mean adaptation progress in different timescales (B: episodes, C: blocks, D: sessions) averaged across all angular transformations. Illustrated are between-subject means and standard errors.

Fig 4A illustrates the mean adaptation of all participants of the intervention group to the increasing angular transformations. On average, the participants adapted to each angular transformation by 9.05° (SD: 0.94°). The successive change of movement directions is confirmed by the significance of the main effect angular transformation in the ANOVA (F(7,161) = 563.85, p<0.001, ŋ^2^_p_ = 0.96). Moreover, the main factors Episode, Block and Session were significant, confirming that adaptation occurred in different timescales (Fig 4). Significant changes between episodes (Fig 4B) confirm relatively quick adaptive changes (F(2,46) = 151.76, p<0.001, ŋ^2^_p_ = 0.87) compared to slower but significant adaptation across blocks (Fig 4C, F(5,115) = 3.64, p = 0.008, ŋ^2^_p_ = 0.14) and sessions (Fig 4D, F(2,46) = 67.59, p<0.001, ŋ^2^_p_ = 0.75). The rate of adaptation changed over time which is confirmed by the significant interactions between episode and block (F(10,230) = 5.04, p<0.001, ŋ^2^_p_ = 0.18) and block and session (F(10,230) = 2.21, p = 0.018, ŋ^2^_p_ = 0.09).

The significance of the main effects angular transformation, episode, block and session confirms that adaptation progressed significantly over time; despite the switching between the adaptation tasks. Further analyses confirm that the participants learned to switch predictively: When exposed to the 100° angular transformation, the participants directed their first movement in each of the four adaptation tasks on average at 60° (SD: 30°). This value differed significantly from the mean direction of the baseline phase (each p<0.001), i.e., the participants aimed their very first movements adequately in the clockwise or counterclockwise direction.

### Sequential adaptation effects

The participants adapted their movements in response to the increasing transformations; however, adaptation was incomplete. A significant interaction of the factor order with the factor angular transformation shows that the performance in the first two adaptation tasks developed better than the performance in the third and fourth adaptation tasks (F(7,161) = 4.66, p = 0.007, ŋ^2^_p_ = 0.17).

**Fig 5:**
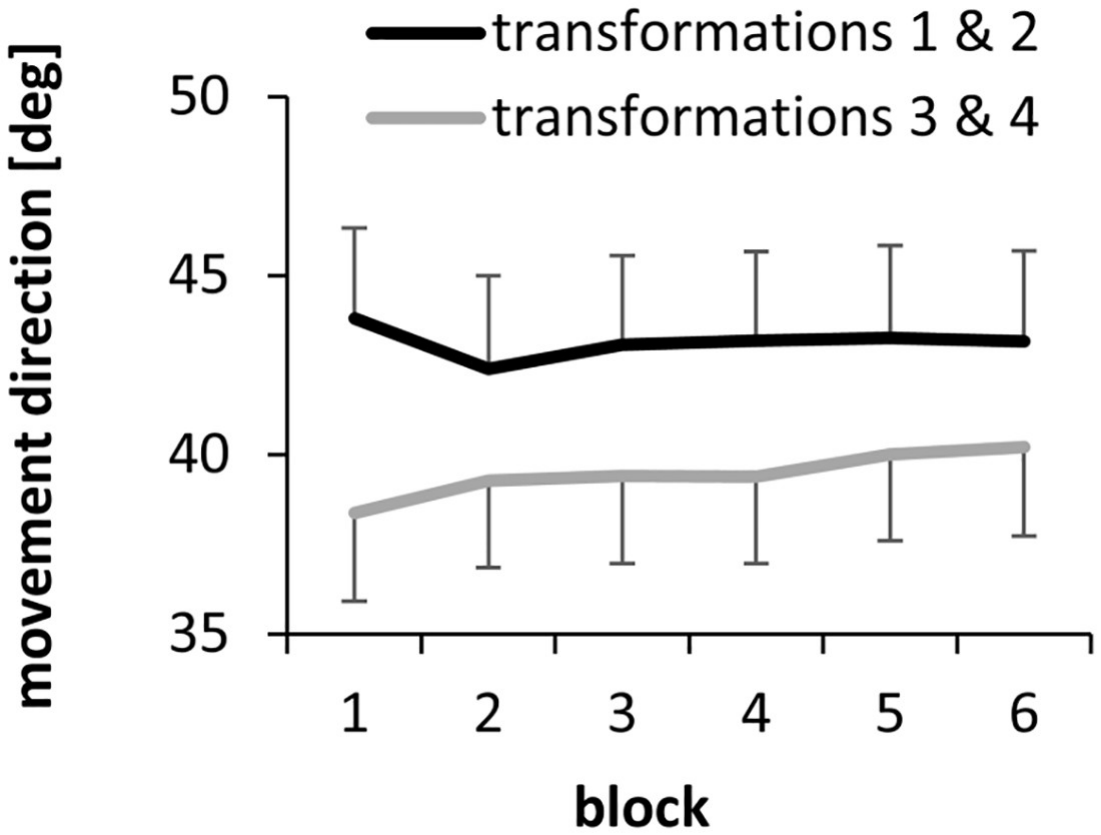
Progress of adaptation across blocks in the first and second compared to the third and fourth adaptation task. 65° represents the expected value for complete adaptation. 0° represents the unadapted expectation. Illustrated are between-subject means and standard errors.

Moreover, the interaction order*block was significant (F(5,115) = 6.07, p<0.001, ŋ^2^_p_ = 0.21). During the third and fourth tasks, the participants significantly improved their performance from block 1 to the other blocks (post-hoc tests: block 1 versus blocks 2 to 6 at least p<0.05). During the first two adaptation tasks, they showed lower performance in the second than in the first block during (post-hoc test: p = 0.003, Fig 5). Concerning the course of adaptation within blocks, the latter differences were larger at the beginning of a block than at the end of a block, i.e., the differences between blocks 1 and 2 were larger in episode 1 than in episodes 2 and 3. This is confirmed by the interaction block*order*episode (F(10,230) = 2.56, p = 0.006, ŋ^2^_p_ = 0.10). The main effect order was not significant.

**Fig 6:**
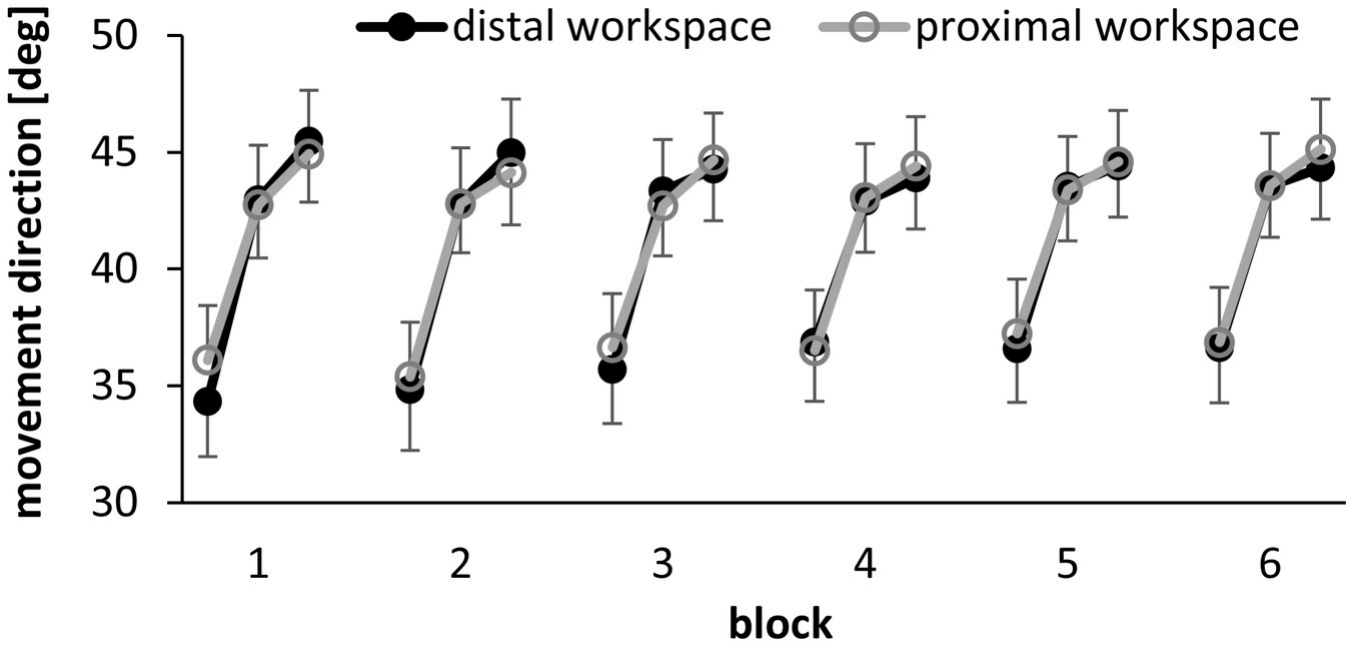
Progress of adaptation across episodes and blocks in the distal and proximal workspace. 65° represents the expected value for complete adaptation. 0° represents the unadapted expectation. Each dot represents the mean and the bars the standard error of all participants of the intervention group in one episode.

Fig 6 illustrates that the performance improved within each block and then decreased at the beginning of the next block (post-hoc comparisons: each p<0.001). This effect differed between workspaces (block*workspace*episode, F(10,230) = 2.61, p = 0.005, ŋ^2^_p_ = 0.10). A post-hoc test revealed significant differences between the workspaces in the first episode of the first block (p<0.001), which confirms that the participants adapted faster in the proximal workspace than in the distal workspace. Furthermore, the interaction of workspace and session was significant (F(2,46) = 6.94, p = 0.002, ŋ^2^_p_ = 0.23). Adaptation progressed quite similar in both workspaces in sessions 1 and 2 (post-hoc tests: all p>0.05). It was significantly better in the proximal than the distal workspace in session 3 (post-hoc tests: p<0.001).

### Cognitive performance changes

All results of the cognitive performance tests are shown in Table 1. An ANOVA of the z-transformed pre-test values neither revealed significant differences between groups nor a test*group interaction (group: F(1,34) = 2.15, p = 0.152, ŋ^2^_p_ = 0.06; test*group: F(11,374) = 1.27, p = 0.245, ŋ^2^_p_ = 0.04). In contrast, ANOVAs on the z-transformed post-pre-changes revealed a significant main effect of group: mean post-pre changes, i.e. the mean cognitive performance changes, were significantly larger in the intervention group than in the passive control group (without the participant with missing data: F(1,33) = 4.32, p = 0.045, ŋ^2^_p_ = 0.12; with all participants but without TMT: (F(1,34) = 4.93, p = 0.033, ŋ^2^_p_ = 0.13). The test*group interaction was not significant (without one participant: F(11,363) = 0.97, p = 0.476, ŋ^2^_p_ = 0.03; without TMT: F(9,306) = 1.10, p = 0.362, ŋ^2^_p_ = 0.03).

**Table 1:** Cognitive performance.

		Intervention group			z	Control group			z
		Pre	Post	Post-Pre		Pre	Post	Post-Pre	
**5 Point Test**	correct patterns	41.54 (8.32)	52.08 (9.28)	-10.54* (6.14)	-0.15* (0.90)	47.42 (11.21)	54.83 (7.49)	-7.42* (7.84)	0.31* (1.15)
	repeated patterns [%]	5.13 (6.05)	3.64 (5.82)	**-1.48** (6.10)	**-0.09** (1.01)	5.36 (4.03)	5.52 (4.52)	**0.16** (6.06)	**0.18** (1.00)
**Stroop Test**	reading words [s]	29.05 (5.31)	26.07 (3.24)	**-2.99** (5.05)	**-0.20** (1.11)	27.25 (4.04)	27.05 (4.70)	**-0.19** (2.71)	**0.41** (0.59)
	naming colors [s]	40.59 (6.69)	37.68 (5.68)	**-2.91** (3.30)	-**0.30** (0.83)	42.67 (7.80)	43.30 (9.14)	**0.63** (4.26)	**0.60** (1.07)
	naming word color [s]	62.75 (11.69)	58.62 (13.04)	**-4.13** (8.35)	**0.01** (1.06)	66.61 (12.38)	62.34 (7.51)	**-4.28** (7.19)	**-0.01** (0.91)
**Trail Making Test**	task A [s]	25.34 (8.85)	20.40 (5.05)	**-4.94** (8.13)	**-0.04** (1.01)	22.72 (6.30)	18.82 (3.51)	**-3.90** (8.14)	**0.08** (1.01)
	task B [s]	40.30 (11.54)	33.05 (9.81)	**-7.61** (11.14)	**-0.13** (0.88)	35.81 (10.11)	33.03 (13.44)	**-2.78** (15.28)	**0.25** (1.20)
**Fair 2 Test**	K	413.22 (93.00)	513.69 (94.79)	-100.46* (88.71)	-0.06* (1.16)	452.61 (57.51)	539.52 (71.11)	-86.91* (45.62)	0.12* (0.60)
**Maze Test**	maze [s]	32.24 (15.52)	23.50 (7.72)	**-8.73** (16.76)	**-0.14** (1.10)	26.39 (6.33)	24.24 (9.66)	**-2.15** (10.96)	**0.29** (0.72)
	pseudo-maze [s]	13.76 (3.88)	11.62 (3.16)	**-2.14** (2.97)	**-0.11** (1.10)	10.83 (1.48)	9.54 (1.67)	**-1.28** (2.06)	**0.21** (0.76)
**Digit Span Test**	correct answers	8.75 (2.01)	9.25 (1.62)	-0.50* (1.29)	-0.09* (1.03)	9.50 (1.68)	9.67 (1.50)	-0.17* (1.19)	0.18* (0.96)
**Number Connection Test**	[s]	60.03 (11.60)	58.26 (11.56)	**-1.77** (7.41)	**0.15** (0.99)	57.89 (6.24)	52.88 (6.37)	**-5.01** (7.35)	**-0.29** (0.99)

Shown are between-subject means (and standard deviations).

z: z-transformed post-pre-changes.

* For these tests, negative post-pre changes reflect performance deteriorations, whereas for all other tests, negative post-pre changes reflect improvements. Therefore, post-pre changes were inverted for better comparability.

### Relation between the cognitive performance changes and the visuomotor adaptation

**Fig 7:**
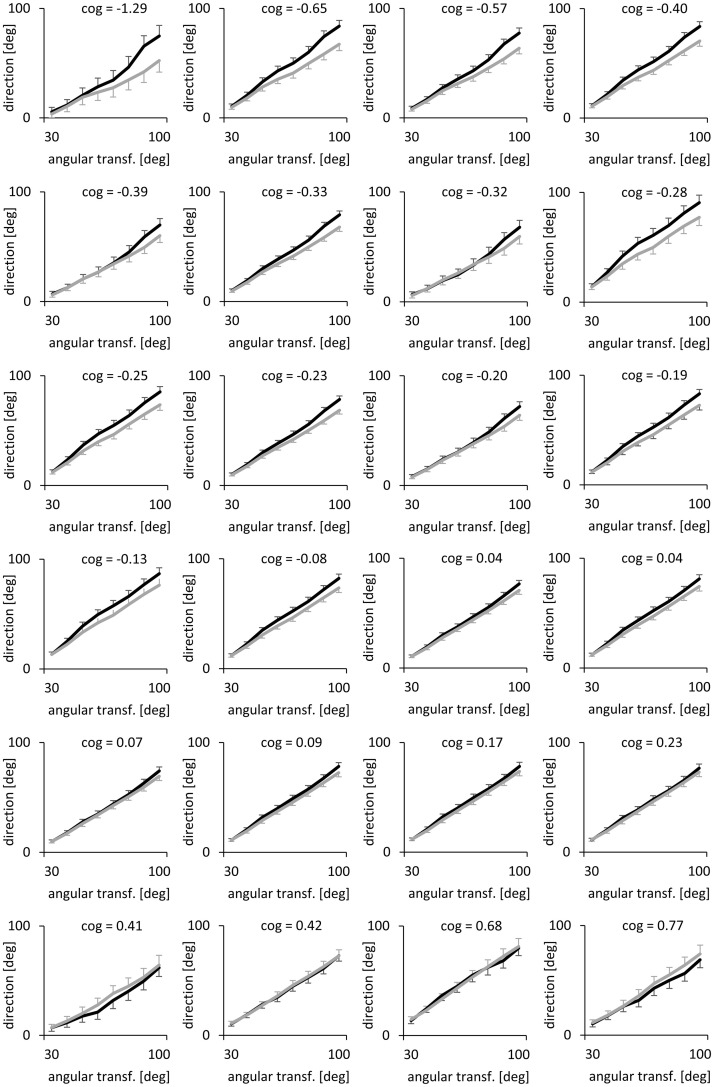
Predictions of the generalized linear model with mean cognitive performance change as a covariate. Predictions of the generalized linear model regarding the performance in the first and second adaptation task (black lines) compared to the third and fourth adaptation task (grey lines) with mean cognitive performance change as a covariate. Each graph shows the values of one participant. Bars represent standard errors of predictions. The size of an angular transformation corresponds to the expected value for complete adaptation. 0° represents the unadapted expectation. "cog" denotes the z-transformed mean cognitive performance change of one participant. As a reference, the mean cognitive performance change of the passive control group might be considered, which was 0.19 (SD: 0.20).

The aim of the next analysis was to find out whether the results from the cognitive performance tests are related to the performance during the visuomotor adaptation intervention. To this end, the mean pre-test-values and the mean cognitive performance changes of each participant of the intervention group were submitted as covariates to the generalized linear model of the adaptation intervention. One significant covariation was found between the mean cognitive performance changes and the interaction angular transformation*order (F(7,154) = 3.22, p = 0.028, ŋ^2^_p_ = 0.13). This covariation can be illustrated based on the prediction values from the generalized linear model. Fig 7 illustrates each participant’s prediction values and standard errors considering their cognitive performance change. The more divergent the performances are in the first and second compared to the third and fourth adaptation tasks, the larger is the cognitive improvement. Mean cognitive performance change was also significant covariate for the interactions block*workspace*episode (F(10,210) = 2.25, p = 0.016, ŋ^2^_p_ = 0.10) and block*order*workspace*episode (F(10,210) = 1.98, p = 0.040, ŋ^2^_p_ = 0.09). Notably, pre-test performance was not a significant covariate for these interactions. Pre-test performance was significant covariate for block (F(5,105) = 3.44, p = 0.022, ŋ^2^_p_ = 0.12), session*block*workspace (F(10,210) = 3.19, p = 0.001, ŋ^2^_p_ = 0.13), block*order*workspace (F(5,105) = 2.31, p = 0.049, ŋ^2^_p_ = 0.10), transformation*order*episode (F(14,294) = 2.23, p = 0.045, ŋ^2^_p_ = 0.10) and transformation*session*workspace*episode (F(28,588) = 1.62, p = 0.033, ŋ^2^_p_ = 0.07).

The mean cognitive performance change was taken as a covariate because it differed significantly between the intervention and the control group and the test*group interaction was not significant. However, it remains unclear whether the mean cognitive performance enhancement was based on an improvement of a single cognitive component inherent to all cognitive tests or an improvement of several cognitive components in parallel. Therefore, a second covariation analysis was performed with a different set of covariates. As this was planned a posteriori, the result was Bonferroni-corrected. The new set of covariates was derived from a factor analysis of the post-pre-changes. The five variables shown in Table 2 met the a priori defined inclusion criteria for the factor analysis. The minimal anti-image correlation was 0.65 (Digit Span Test), and the minimal commonality was 0.75 (pseudo-maze). The Kaiser-Meyer-Olkin-criterion was 0.74, and the Bartlett-Test for sphericity was significant (X^2^ = 41.60, p<0.001). The inspection of the scree plot revealed an inflection point below the second factor. Both factors had Eigenvalues larger than 1 and therefore were extracted.

**Table 2:** Factor analysis of post-pre changes in the cognitive performance tests.

Variable	Factor 1	Factor 2
Stroop Test: reading words [s]	**0.90**	0.05
Stroop Test: naming the color of bars [s]	**0.86**	0.22
Trail Making Test: task A [s]	**0.76**	0.42
Maze Test: pseudo-maze [s]	0.06	**0.92**
Digit Span Test: correct answers	0.39	**0.79**
Eigenvalue	2.93	1.03
% explained variance	58.56	20.67

Applied was the principal component method with standardized varimax rotation. Illustrated are factors with eigenvalues larger than 1. Factor loadings larger than 0.7 are highlighted for clarity.

The result of the factor analysis is shown in Table 2. Each of the five variables can be assigned more clearly to the one factor than to the other. The Stroop Test measures and the Trail Making Test predominantly load on factor 1, and the measures from the Maze and the Digit Span Test predominantly load on factor 2. The estimation of the stability of the factor structure resulted in 0.80 when the factor loading of 0.76 from the Trail Making Test is considered as the lowest threshold and thus matches the minimal value proposed by Bortz and Schuster [44].

With the individual factor scores, the significant covariation with the interaction angular transformation*order was replicated. Notably, significant covariations with this interaction were achieved for both extracted factors (factor 1: F(7,133) = 4.55, p = 0.008, ŋ^2^_p_ = 0.19; factor 2: F(7,133) = 3.33, p = 0.04, ŋ^2^_p_ = 0.15). As these factors are orthogonal, these results point to covariations of two independent variance components with the visuomotor adaptation intervention. Factor 1 was also covariate for block (F(5,95) = 3.13, p = 0.024, ŋ^2^_p_ = 0.14), transformation*block*episode (F(70,1330) = 1.69, p = 0.008, ŋ^2^_p_ = 0.08), transformation*session*order*episode (F(28,532) = 1.75, p = 0.022, ŋ^2^_p_ = 0.08). Factor 2 was also significant covariate for transformation*session (F(14,266) = 1.92, p = 0.048, ŋ^2^_p_ = 0.09).

## Discussion

The present study investigated whether multiple adaptations to visuomotor transformations significantly affect cognitive performance. The results indicate that the participants of the intervention group adapted to all transformations and significantly increased their cognitive performance compared to a passive control group.

### Adaptation

The participants of the intervention group adapted to each angular transformation by about 9°. The adaptation to the 30° transformation developed on a lower level than previously reported by two studies that had used the same experimental apparatus [8, 15]. This finding might be explained by the fact that the participants performed four adaptation tasks alternatingly. Albert et al. [45] have shown that changes of the size or the sign of angular transformations enhance residual adaptation errors. The different demands of the four adaptation tasks and the increasing angular transformations might have had a similar effect in the present study.

The overall adaptation illustrated by Fig 4A is the averaged performance at the four different adaptation tasks; the participants adapted in all four tasks concurrently. Concurrent adaptation to four visuomotor transformations was also reported by Thomas and Bock [36], whose participants performed bilateral hand training; i.e., each hand adapted concurrently to two visuomotor transformations. The participants of the present study adapted in all tasks with one hand only. The switching between adaptation tasks performed with one hand typically leads to interference when the tasks require the adaptation of movement directions to opposite directions in the same workspace [17, 37, 46]. This might be another explanation for the observed incomplete adaptation.

Bastian [47] and Wolpert et al. [48] argued that the learning of switching between opposite directed transformations is driven by a different mechanism than the adaptation to a single transformation. The switching needs much more trials to be learned [23, 49]. In the present study, clockwise and counterclockwise transformations switched 288 times, which seemed sufficient for the participants to learn the switching partially. This is shown by the analysis of the very first movements at the 100° transformations, which differed from the mean error of the baseline phase by about 60°.

### Cognitive performance changes

The main goal of the present study was to investigate whether visuomotor adaptations improve cognitive performance. Mean post-pre changes in the cognitive performance tests differed significantly between the intervention and the control group, indicating a causal relationship between sensorimotor adaptation training and cognitive performance.

Ten from twelve test measures showed larger improvements for the intervention than for the control group on a descriptive level. The test*group interaction was not significant; thus, it could not be shown that the post-pre changes differed significantly between the neuropsychological tests. This either suggests an effect of the intervention on a very basal cognitive competence, which affects the performance in nearly all tests, or a similar effect on several cognitive functions in parallel. The factor analysis, which yielded two orthogonal, i.e., independent, factors, indicates that at least two cognitive components had developed in parallel (Table 2).

Factor 1 represents the shared variance of two performance measures from the Stroop Test, one of the Trail Making Test and the Digit Span Test. An interpretation for this factor might be derived from Baeumler [33], who had developed the version of the Stroop Test used in the present study. He described a global cognitive factor relevant for all subtests of the Stroop Test. Baeumler [33] interpreted this factor as self-paced action speed, mental agility and vigilance. Since this factor correlates with various other abilities, like attentional control and vigilance, comprehension speed, fluency, and memory tests, but not with reaction time tests and cognitive decision-making [33], the shared variance of the Stroop Test, the Trail Making Test and the Digit Span Test seem to be plausible, because these tests also measure some of the afore mentioned abilities [28, 30, 33]. Correlations between the Trail Making Test A, the Stroop Test as well as the Digit Span Test have been reported by other authors [29, 30, 50]. Following the interpretation from Salthouse [29] and Sanchez et al. [30], the factor could reflect perceptual speed or processing speed and fluid cognitive abilities as part of the executive functions. Noteworthy, fluency is also assessed by the Five Point Test in the present study, in which the participants of the intervention group scored higher than the participants of the passive control group.

Factor 2 was predominantly loaded by the Maze Test and by the Digit Span Test. Schmitz [15] also reported a covariation between a factor loaded by the Maze Test (factor loading 0.88) and the Digit Span Test (factor loading of 0.58) with the performance in two successive adaptation tasks. Therefore, this result is plausible. Maze-traversing requires spatial-cognitive operations [35, 51] and the Digit Span Test measures explicit working memory resources [34]. Thus, factor 2 might reflect an improvement of such working memory components, which are involved in the memory of visuospatial traces. Spatial memory capacities seem to play an important role during sensorimotor learning, as highlighted by studies from Seidler et al. [6] and Sidarta et al. [52]. Unfortunately, neither visuospatial nor somatosensory working memory has been assessed directly by the present tests. Therefore, this is recommended for future studies.

Anderson [53] presented a conceptual framework for executive control based on four interrelated domains: cognitive flexibility, goal setting, attentional control and information processing. The two factors found in this study can be assigned to two domains of this concept. Factor 1 would be assigned to information processing, which according to Anderson [53] includes speed of processing and fluency. Factor 2 would be assigned to the domain of cognitive flexibility, which according to the authors also includes working memory. The cognitive abilities inhibition, selective attention, perseveration, divided attention, and flexibility, considered relevant in other adaptation studies, can also be attributed to Anderson’s framework, namely to the domains attentional control and cognitive flexibility [3–4, 10, 13–15]. According to this interpretation, adaptation would be related to executive control.

An ANCOVA was conducted to detect possible relationships between cognitive performance changes and factors of the adaptation tasks. It should be noted that a significant covariation between variables provides information about shared variance but does not allow causal attribution. The mean cognitive performance change was a significant covariate for adaptation effects of the intervention group confirming that the mean cognitive performance change shared variance with specific effects of the adaptation tasks. According to the taxonomy of Cohen [54], the effect sizes reflect medium to large effects. The largest effects were observed for the covariations of factor 1 and factor 2 with the interaction angular transformation*order. As illustrated by Fig 7, the cognitive performance changes seem to be related to the performance differences between successive adaptation tasks. That means that it is not the participants who adapt best who have the largest cognitive improvements, but those who show the largest performance differences between tasks. The performance difference might be interpreted as interference between successive adaptations tasks. A larger degree of interference might have represented a stronger stimulus for cognitive functions, which in turn developed more strongly. Notably, also for the participants with the larger cognitive performance enhancements, the interference persisted until the end of the intervention.

With the exception of the covariations with the factor block, all covariations involved at least one of the factors angular transformation, order or workspace. This might indicate that the increasing task demands as well as the switching or the interference between tasks were relevant for the increase in cognitive performance.

In summary, the present study observed cognitive performance improvements in the intervention compared to the control group. Covariation analyses indicate a possible relationship between the mean cognitive performance change and specific components of the adaptation intervention. Whether these covariations reflect causal effects needs to be investigated in future studies.

## Supporting information

S1 Data (ZIP)
